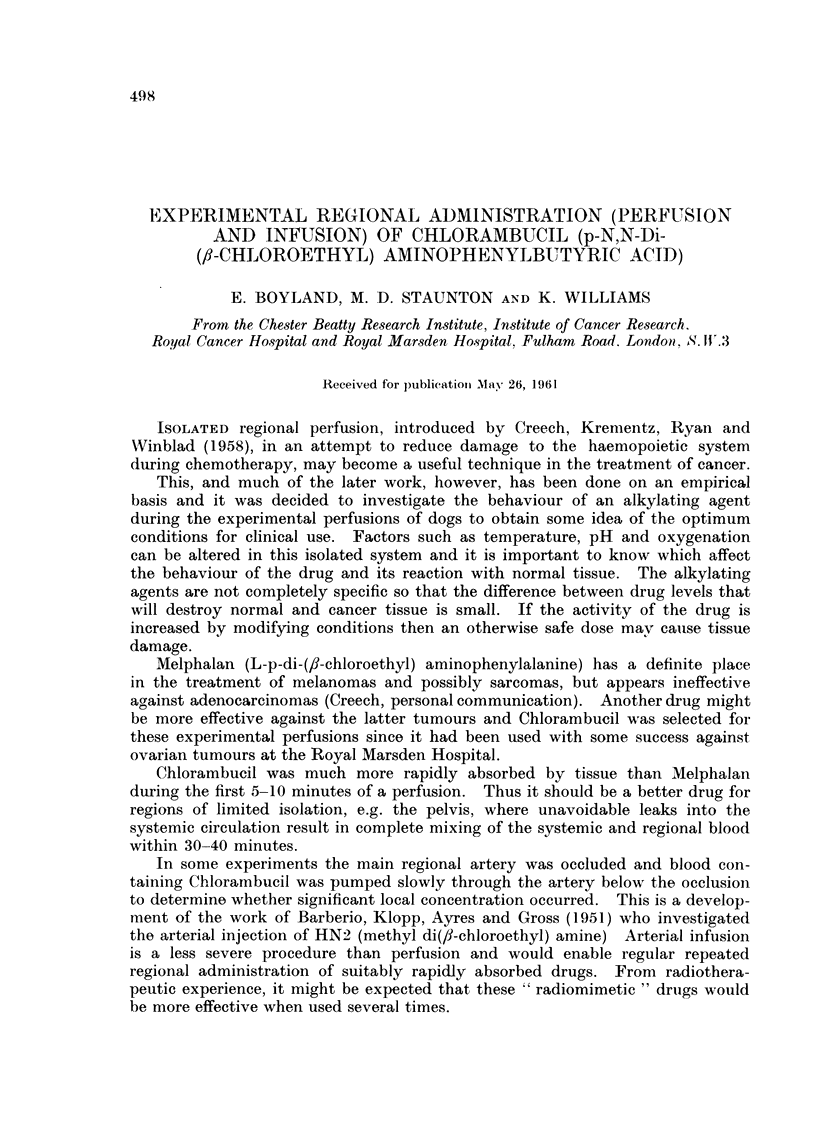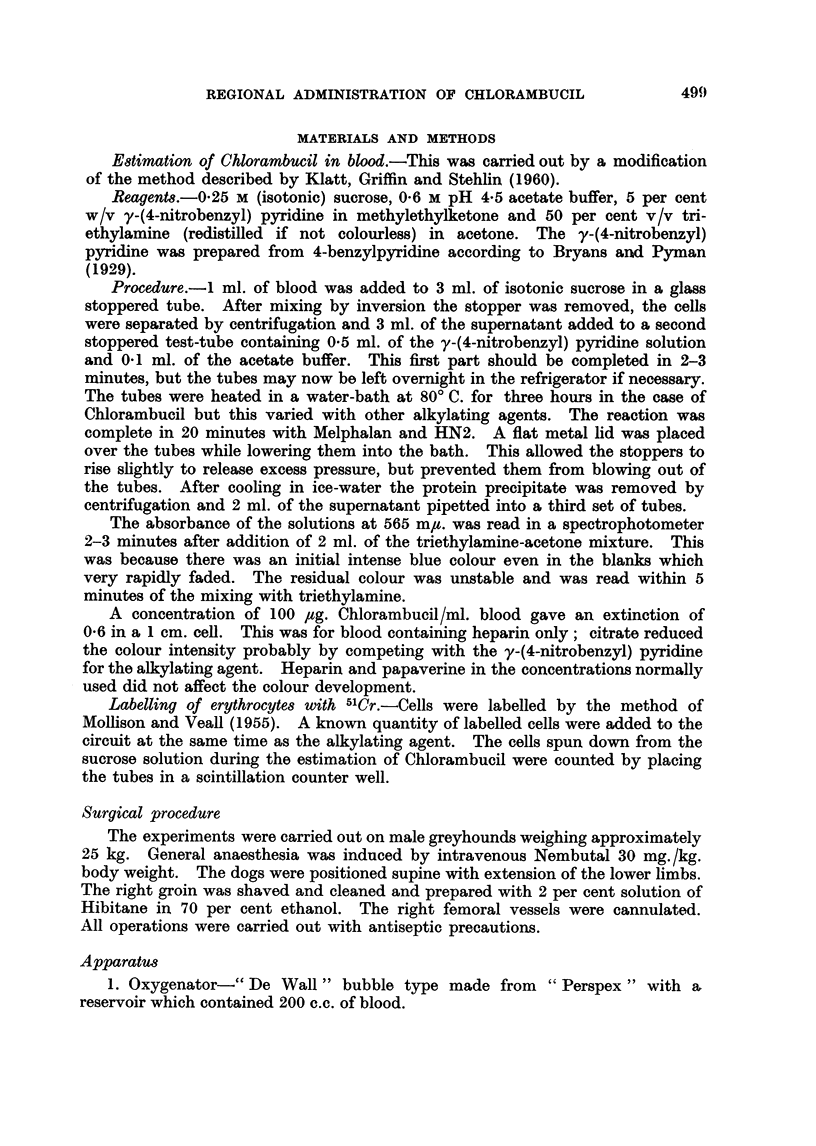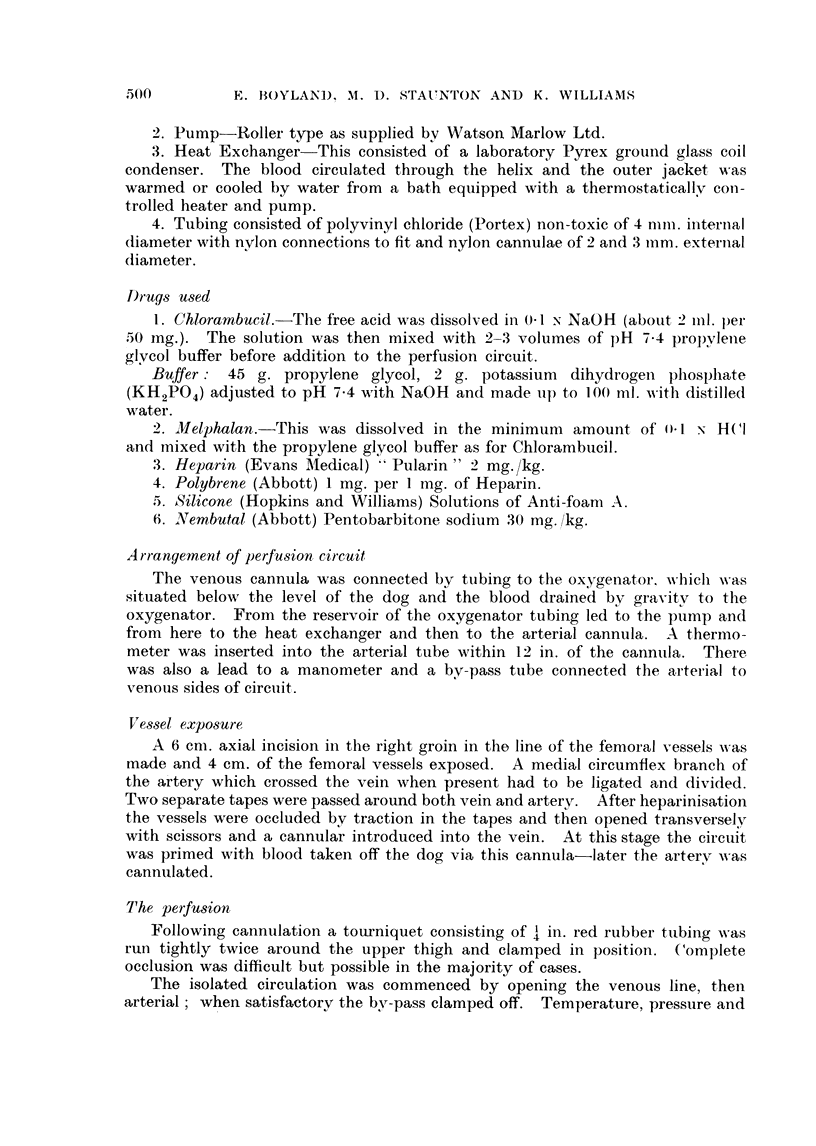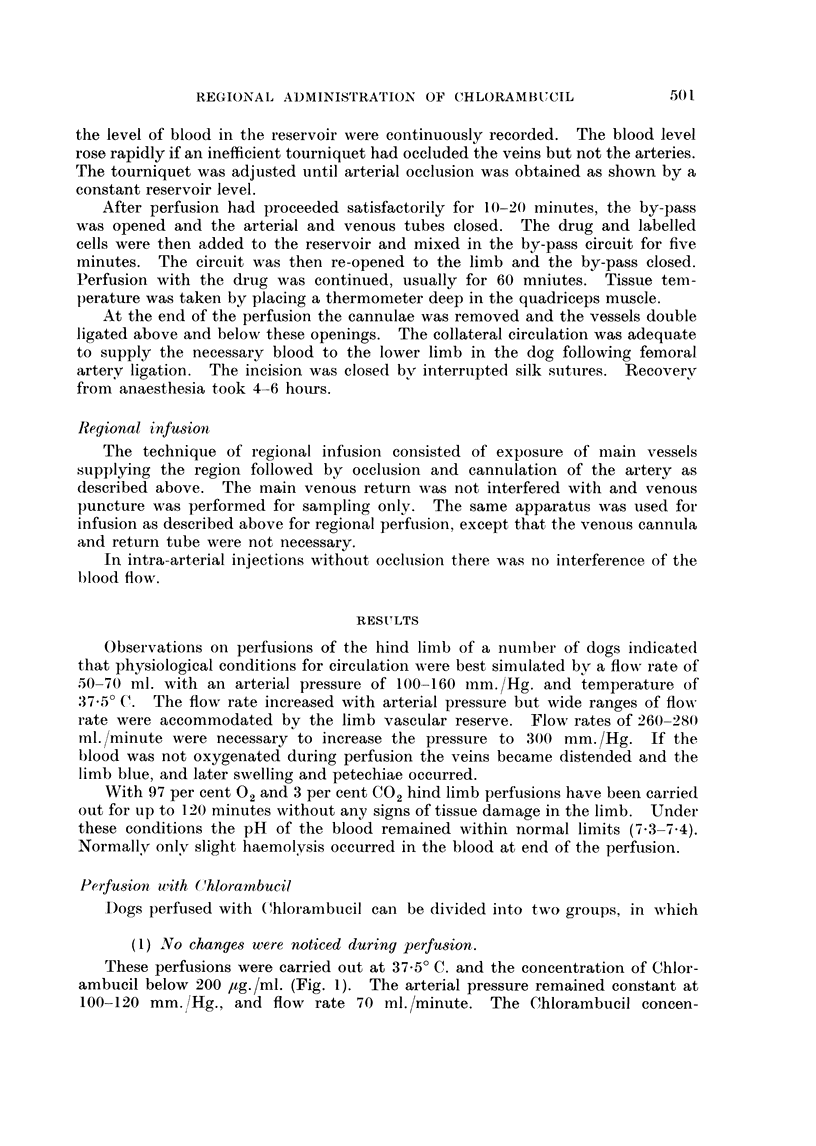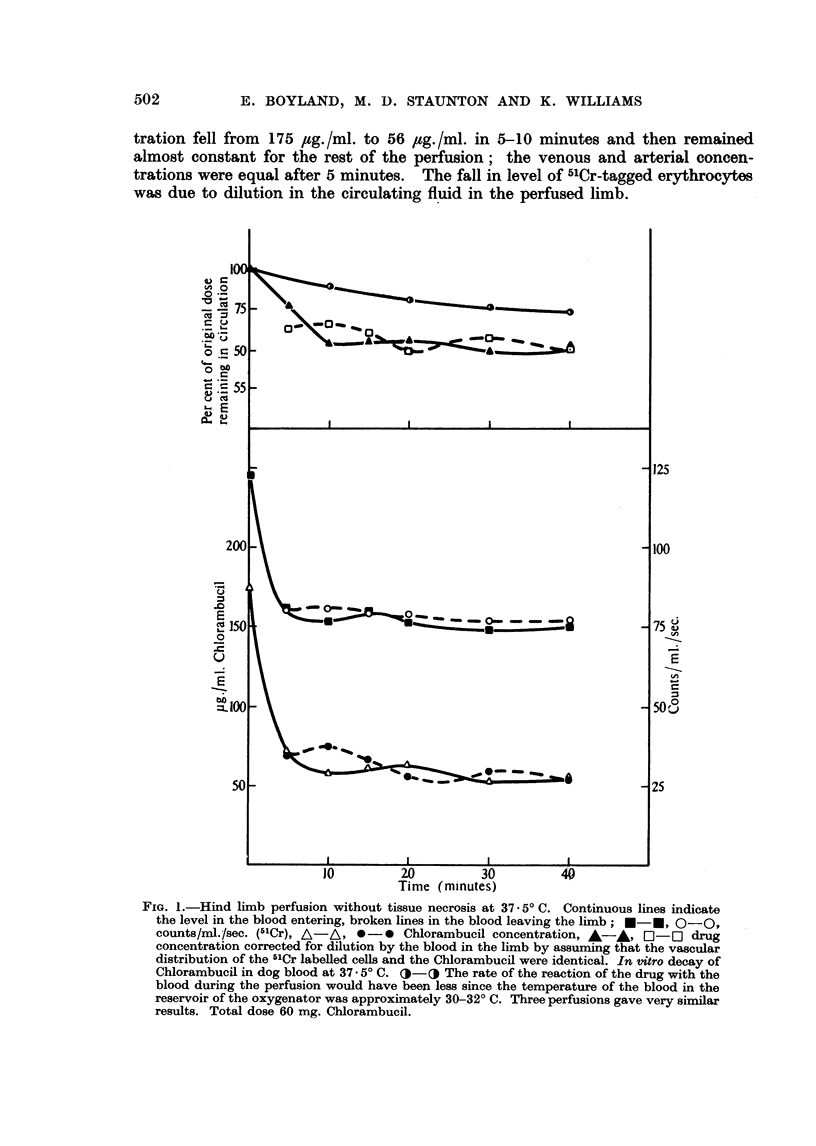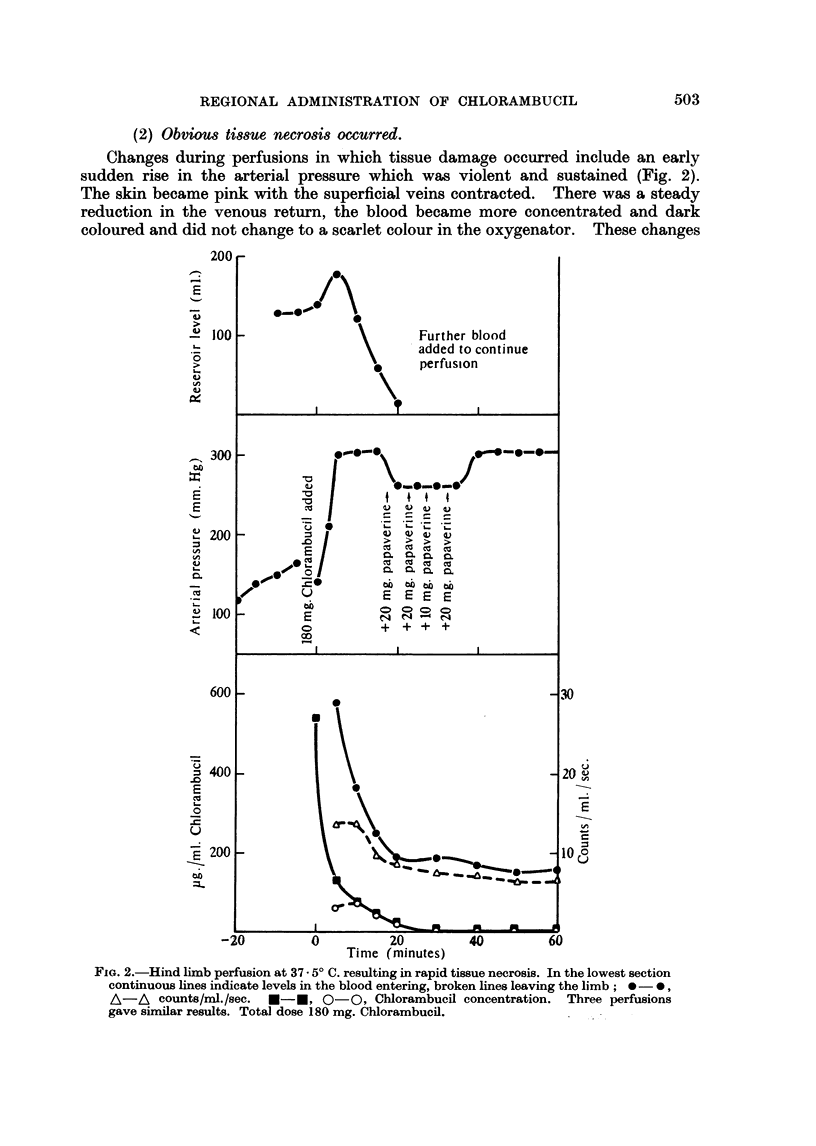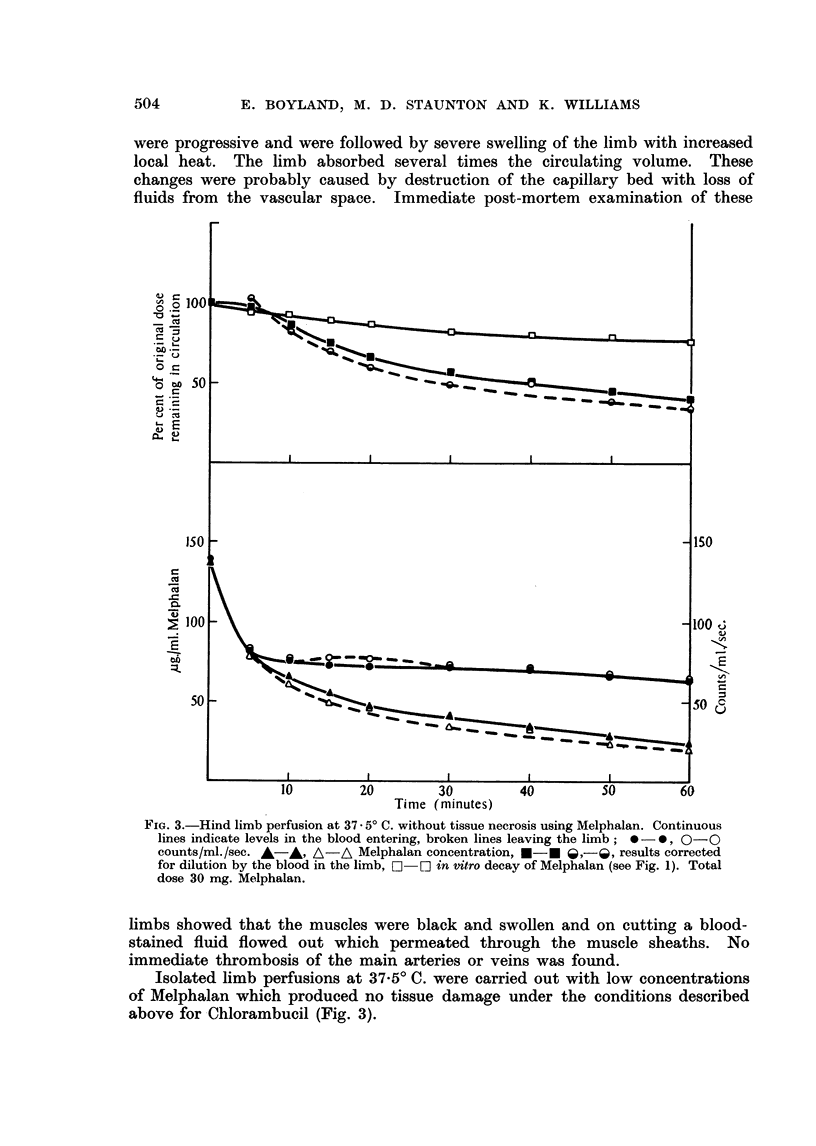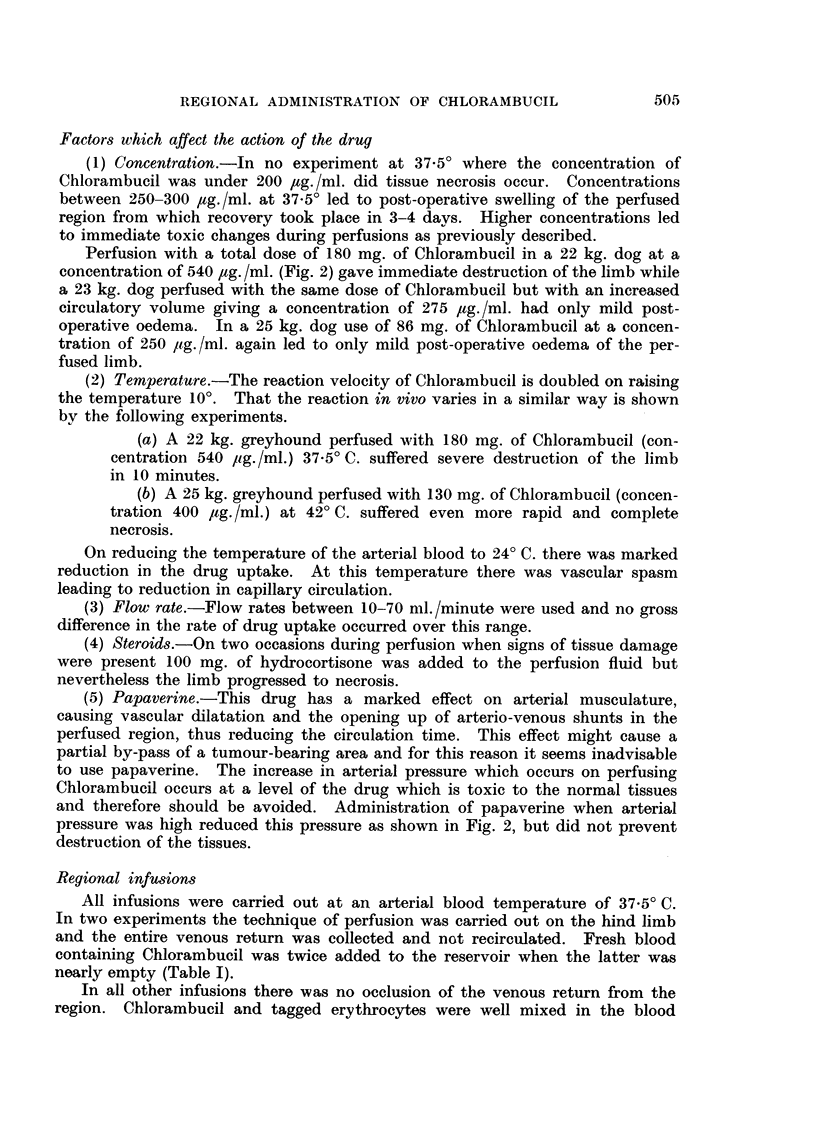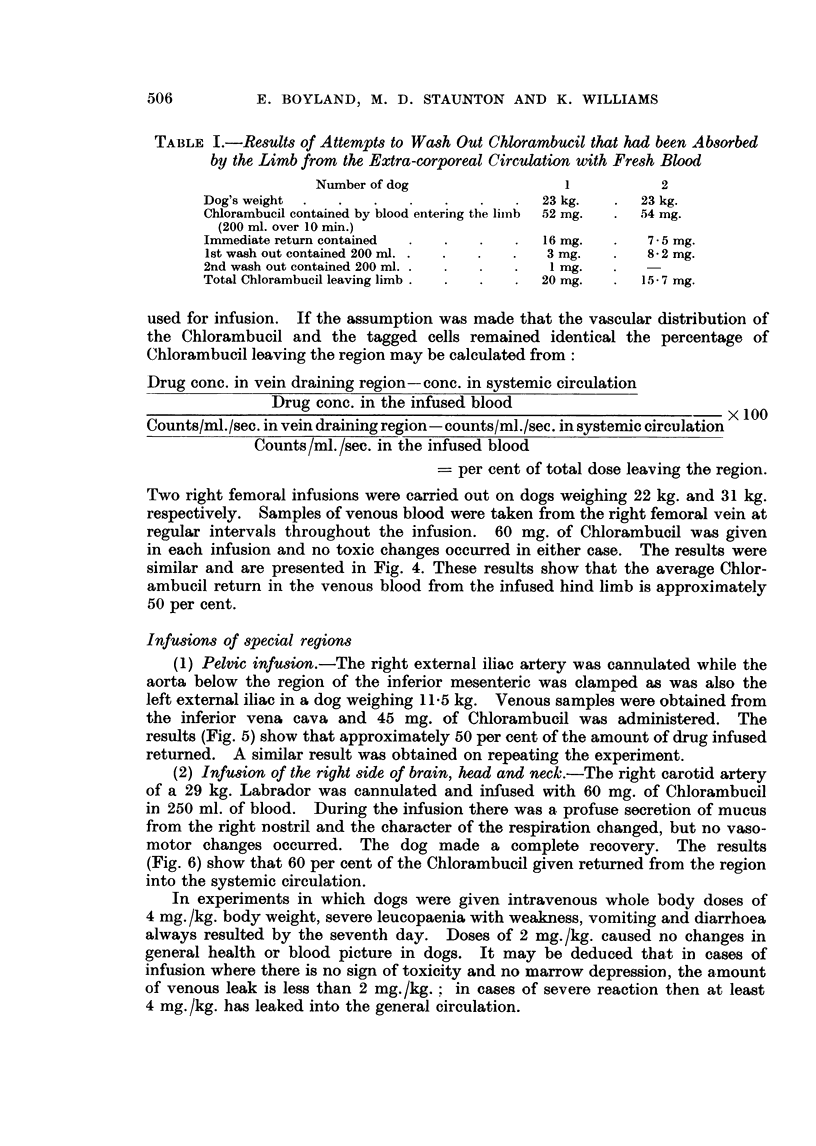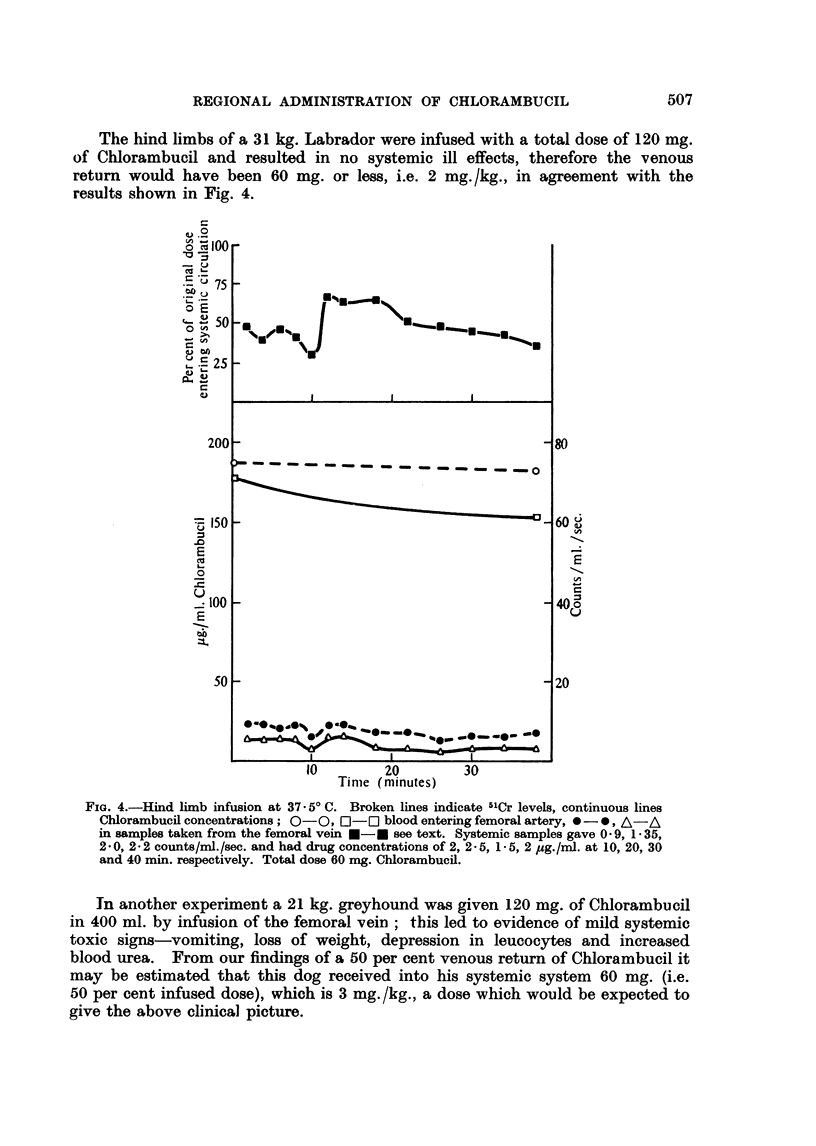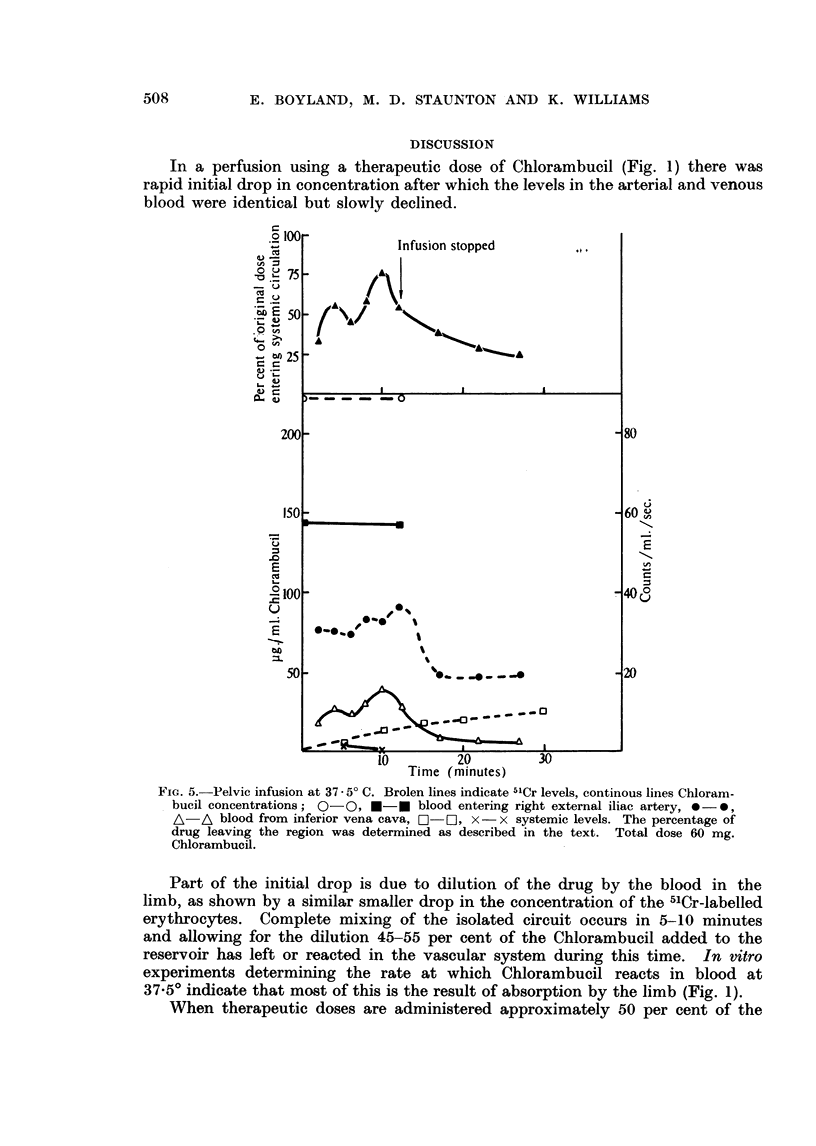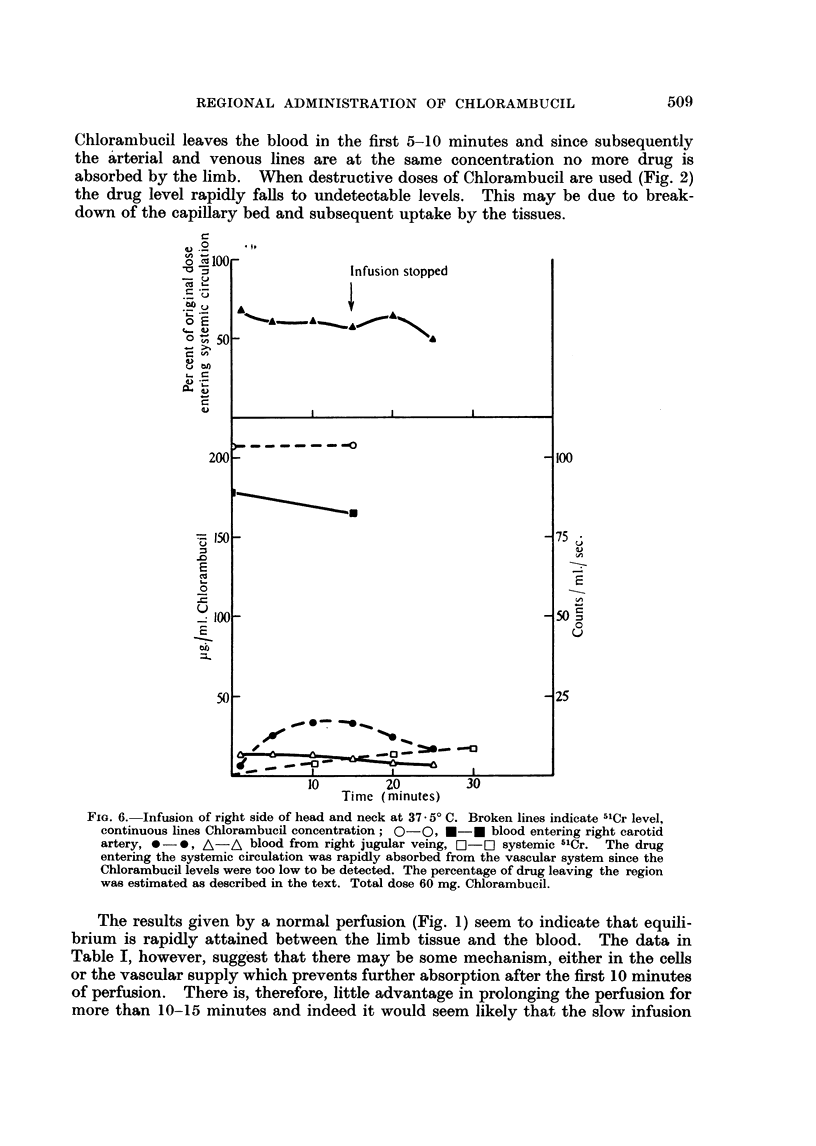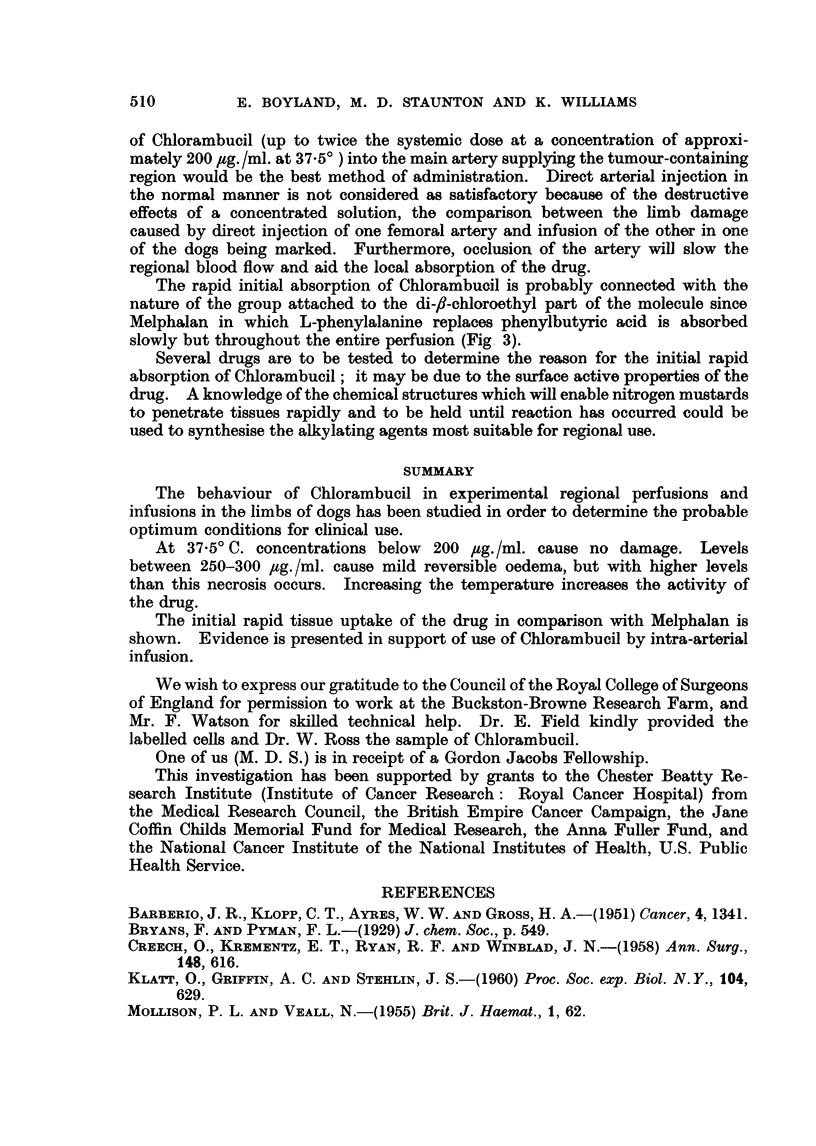# Experimental Regional Administration (Perfusion and Infusion) of Chlorambucil (p-N, N-Di-(β-Chloroethyl) Aminophenylbutyric Acid)

**DOI:** 10.1038/bjc.1961.60

**Published:** 1961-09

**Authors:** E. Boyland, M. D. Staunton, K. Williams


### EXPERIMENTAL REGIONAL ADMINISTRATION (PERFUSION AND INFUSION) OF CHLORAMBUCIL (p-N,N-Di-($\beta$-CHLOROETHYL) AMINOPHENYLBUTYRIC ACID)

E. BOYLAND, M. D. STAUNTON AND K. WILLIAMS

*From the Chester Beatty Research Institute, Institute of Cancer Research, Royal Cancer Hospital and Royal Marsden Hospital, Fulham Road, London, S.W.3*

Received for publication May 26, 1961

ISOLATED regional perfusion, introduced by Creech, Krementz, Ryan and Winblad (1958), in an attempt to reduce damage to the haemopoietic system during chemotherapy, may become a useful technique in the treatment of cancer.

This, and much of the later work, however, has been done on an empirical basis and it was decided to investigate the behaviour of an alkylating agent during the experimental perfusions of dogs to obtain some idea of the optimum conditions for clinical use. Factors such as temperature, pH and oxygenation can be altered in this isolated system and it is important to know which affect the behaviour of the drug and its reaction with normal tissue. The alkylating agents are not completely specific so that the difference between drug levels that will destroy normal and cancer tissue is small. If the activity of the drug is increased by modifying conditions then an otherwise safe dose may cause tissue damage.

Melphalan (L-p-di-($\beta$-chloroethyl) aminophenylalanine) has a definite place in the treatment of melanomas and possibly sarcomas, but appears ineffective against adenocarcinomas (Creech, personal communication). Another drug might be more effective against the latter tumours and Chlorambucil was selected for these experimental perfusions since it had been used with some success against ovarian tumours at the Royal Marsden Hospital.

Chlorambucil was much more rapidly absorbed by tissue than Melphalan during the first 5–10 minutes of a perfusion. Thus it should be a better drug for regions of limited isolation, e.g. the pelvis, where unavoidable leaks into the systemic circulation result in complete mixing of the systemic and regional blood within 30–40 minutes.

In some experiments the main regional artery was occluded and blood containing Chlorambucil was pumped slowly through the artery below the occlusion to determine whether significant local concentration occurred. This is a development of the work of Barberio, Klopp, Ayres and Gross (1951) who investigated the arterial injection of HN2 (methyl di($\beta$-chloroethyl) amine) Arterial infusion is a less severe procedure than perfusion and would enable regular repeated regional administration of suitably rapidly absorbed drugs. From radiotherapeutic experience, it might be expected that these “ radiomimetic ” drugs would be more effective when used several times.

### MATERIALS AND METHODS

*Estimation of Chlorambucil in blood.*—This was carried out by a modification of the method described by Klatt, Griffin and Stehlin (1960).

*Reagents.*—0·25 M (isotonic) sucrose, 0·6 M pH 4·5 acetate buffer, 5 per cent w/v $\gamma$-(4-nitrobenzyl) pyridine in methylethylketone and 50 per cent v/v triethylamine (redistilled if not colourless) in acetone. The $\gamma$-(4-nitrobenzyl) pyridine was prepared from 4-benzylpyridine according to Bryans and Pyman (1929).

*Procedure.*—1 ml. of blood was added to 3 ml. of isotonic sucrose in a glass stoppered tube. After mixing by inversion the stopper was removed, the cells were separated by centrifugation and 3 ml. of the supernatant added to a second stoppered test-tube containing 0·5 ml. of the $\gamma$-(4-nitrobenzyl) pyridine solution and 0·1 ml. of the acetate buffer. This first part should be completed in 2–3 minutes, but the tubes may now be left overnight in the refrigerator if necessary. The tubes were heated in a water-bath at 80° C. for three hours in the case of Chlorambucil but this varied with other alkylating agents. The reaction was complete in 20 minutes with Melphalan and HN2. A flat metal lid was placed over the tubes while lowering them into the bath. This allowed the stoppers to rise slightly to release excess pressure, but prevented them from blowing out of the tubes. After cooling in ice-water the protein precipitate was removed by centrifugation and 2 ml. of the supernatant pipetted into a third set of tubes.

The absorbance of the solutions at 565 m$\mu$. was read in a spectrophotometer 2–3 minutes after addition of 2 ml. of the triethylamine-acetone mixture. This was because there was an initial intense blue colour even in the blanks which very rapidly faded. The residual colour was unstable and was read within 5 minutes of the mixing with triethylamine.

A concentration of 100 $\mu$g. Chlorambucil/ml. blood gave an extinction of 0·6 in a 1 cm. cell. This was for blood containing heparin only ; citrate reduced the colour intensity probably by competing with the $\gamma$-(4-nitrobenzyl) pyridine for the alkylating agent. Heparin and papaverine in the concentrations normally used did not affect the colour development.

*Labelling of erythrocytes with* $^{51}Cr$.—Cells were labelled by the method of Mollison and Veall (1955). A known quantity of labelled cells were added to the circuit at the same time as the alkylating agent. The cells spun down from the sucrose solution during the estimation of Chlorambucil were counted by placing the tubes in a scintillation counter well.

### *Surgical procedure*

The experiments were carried out on male greyhounds weighing approximately 25 kg. General anaesthesia was induced by intravenous Nembutal 30 mg./kg. body weight. The dogs were positioned supine with extension of the lower limbs. The right groin was shaved and cleaned and prepared with 2 per cent solution of Hibitane in 70 per cent ethanol. The right femoral vessels were cannulated. All operations were carried out with antiseptic precautions.

### *Apparatus*

1. Oxygenator—" De Wall " bubble type made from " Perspex " with a reservoir which contained 200 c.c. of blood.

2. Pump—Roller type as supplied by Watson Marlow Ltd.
3. Heat Exchanger—This consisted of a laboratory Pyrex ground glass coil condenser. The blood circulated through the helix and the outer jacket was warmed or cooled by water from a bath equipped with a thermostatically controlled heater and pump.
4. Tubing consisted of polyvinyl chloride (Portex) non-toxic of 4 mm. internal diameter with nylon connections to fit and nylon cannulae of 2 and 3 mm. external diameter.

*Drugs used*

1. *Chlorambucil.*—The free acid was dissolved in 0·1 N NaOH (about 2 ml. per 50 mg.). The solution was then mixed with 2–3 volumes of pH 7·4 propylene glycol buffer before addition to the perfusion circuit.

*Buffer:* 45 g. propylene glycol, 2 g. potassium dihydrogen phosphate ($KH_2PO_4$) adjusted to pH 7·4 with NaOH and made up to 100 ml. with distilled water.

2. *Melphalan.*—This was dissolved in the minimum amount of 0·1 N HCl and mixed with the propylene glycol buffer as for Chlorambucil.
3. *Heparin* (Evans Medical) "Pularin" 2 mg./kg.
4. *Polybrene* (Abbott) 1 mg. per 1 mg. of Heparin.
5. *Silicone* (Hopkins and Williams) Solutions of Anti-foam A.
6. *Nembutal* (Abbott) Pentobarbitone sodium 30 mg./kg.

*Arrangement of perfusion circuit*

The venous cannula was connected by tubing to the oxygenator, which was situated below the level of the dog and the blood drained by gravity to the oxygenator. From the reservoir of the oxygenator tubing led to the pump and from here to the heat exchanger and then to the arterial cannula. A thermometer was inserted into the arterial tube within 12 in. of the cannula. There was also a lead to a manometer and a by-pass tube connected the arterial to venous sides of circuit.

*Vessel exposure*

A 6 cm. axial incision in the right groin in the line of the femoral vessels was made and 4 cm. of the femoral vessels exposed. A medial circumflex branch of the artery which crossed the vein when present had to be ligated and divided. Two separate tapes were passed around both vein and artery. After heparinisation the vessels were occluded by traction in the tapes and then opened transversely with scissors and a cannular introduced into the vein. At this stage the circuit was primed with blood taken off the dog via this cannula—later the artery was cannulated.

*The perfusion*

Following cannulation a tourniquet consisting of ¼ in. red rubber tubing was run tightly twice around the upper thigh and clamped in position. Complete occlusion was difficult but possible in the majority of cases.

The isolated circulation was commenced by opening the venous line, then arterial ; when satisfactory the by-pass clamped off. Temperature, pressure and the level of blood in the reservoir were continuously recorded. The blood level rose rapidly if an inefficient tourniquet had occluded the veins but not the arteries. The tourniquet was adjusted until arterial occlusion was obtained as shown by a constant reservoir level.

After perfusion had proceeded satisfactorily for 10–20 minutes, the by-pass was opened and the arterial and venous tubes closed. The drug and labelled cells were then added to the reservoir and mixed in the by-pass circuit for five minutes. The circuit was then re-opened to the limb and the by-pass closed. Perfusion with the drug was continued, usually for 60 mniutes. Tissue temperature was taken by placing a thermometer deep in the quadriceps muscle.

At the end of the perfusion the cannulae was removed and the vessels double ligated above and below these openings. The collateral circulation was adequate to supply the necessary blood to the lower limb in the dog following femoral artery ligation. The incision was closed by interrupted silk sutures. Recovery from anaesthesia took 4–6 hours.

*Regional infusion*

The technique of regional infusion consisted of exposure of main vessels supplying the region followed by occlusion and cannulation of the artery as described above. The main venous return was not interfered with and venous puncture was performed for sampling only. The same apparatus was used for infusion as described above for regional perfusion, except that the venous cannula and return tube were not necessary.

In intra-arterial injections without occlusion there was no interference of the blood flow.

### RESULTS

Observations on perfusions of the hind limb of a number of dogs indicated that physiological conditions for circulation were best simulated by a flow rate of 50–70 ml. with an arterial pressure of 100–160 mm./Hg. and temperature of 37·5° C. The flow rate increased with arterial pressure but wide ranges of flow rate were accommodated by the limb vascular reserve. Flow rates of 260–280 ml./minute were necessary to increase the pressure to 300 mm./Hg. If the blood was not oxygenated during perfusion the veins became distended and the limb blue, and later swelling and petechiae occurred.

With 97 per cent $O_2$ and 3 per cent $CO_2$ hind limb perfusions have been carried out for up to 120 minutes without any signs of tissue damage in the limb. Under these conditions the pH of the blood remained within normal limits (7·3–7·4). Normally only slight haemolysis occurred in the blood at end of the perfusion.

*Perfusion with Chlorambucil*

Dogs perfused with Chlorambucil can be divided into two groups, in which

(1) *No changes were noticed during perfusion.*

These perfusions were carried out at 37·5° C. and the concentration of Chlorambucil below 200 $\mu$g./ml. (Fig. 1). The arterial pressure remained constant at 100–120 mm./Hg., and flow rate 70 ml./minute. The Chlorambucil concentration fell from 175 μg./ml. to 56 μg./ml. in 5–10 minutes and then remained almost constant for the rest of the perfusion; the venous and arterial concentrations were equal after 5 minutes. The fall in level of $^{51}$Cr-tagged erythrocytes was due to dilution in the circulating fluid in the perfused limb.

FIG. 1.—Hind limb perfusion without tissue necrosis at 37·5° C. Continuous lines indicate the level in the blood entering, broken lines in the blood leaving the limb; ■—■, ○—○, counts/ml./sec. ($^{51}$Cr), △—△, ●—● Chlorambucil concentration, ▲—▲, □—□ drug concentration corrected for dilution by the blood in the limb by assuming that the vascular distribution of the $^{51}$Cr labelled cells and the Chlorambucil were identical. *In vitro* decay of Chlorambucil in dog blood at 37·5° C. ◑—◑ The rate of the reaction of the drug with the blood during the perfusion would have been less since the temperature of the blood in the reservoir of the oxygenator was approximately 30–32° C. Three perfusions gave very similar results. Total dose 60 mg. Chlorambucil.

(2) *Obvious tissue necrosis occurred.*

Changes during perfusions in which tissue damage occurred include an early sudden rise in the arterial pressure which was violent and sustained (Fig. 2). The skin became pink with the superficial veins contracted. There was a steady reduction in the venous return, the blood became more concentrated and dark coloured and did not change to a scarlet colour in the oxygenator. These changes

Fig. 2.—Hind limb perfusion at 37·5° C. resulting in rapid tissue necrosis. In the lowest section continuous lines indicate levels in the blood entering, broken lines leaving the limb ; ●—●, △—△ counts/ml./sec. ■—■, ○—○, Chlorambucil concentration. Three perfusions gave similar results. Total dose 180 mg. Chlorambucil.

were progressive and were followed by severe swelling of the limb with increased local heat. The limb absorbed several times the circulating volume. These changes were probably caused by destruction of the capillary bed with loss of fluids from the vascular space. Immediate post-mortem examination of these

FIG. 3.—Hind limb perfusion at 37·5° C. without tissue necrosis using Melphalan. Continuous lines indicate levels in the blood entering, broken lines leaving the limb; ●—●, ○—○ counts/ml./sec. ▲—▲, △—△ Melphalan concentration, ■—■ ◒,—◒, results corrected for dilution by the blood in the limb, □—□ *in vitro* decay of Melphalan (see Fig. 1). Total dose 30 mg. Melphalan.

limbs showed that the muscles were black and swollen and on cutting a blood-stained fluid flowed out which permeated through the muscle sheaths. No immediate thrombosis of the main arteries or veins was found.

Isolated limb perfusions at 37·5° C. were carried out with low concentrations of Melphalan which produced no tissue damage under the conditions described above for Chlorambucil (Fig. 3).

*Factors which affect the action of the drug*

(1) *Concentration.*—In no experiment at 37·5° where the concentration of Chlorambucil was under 200 μg./ml. did tissue necrosis occur. Concentrations between 250–300 μg./ml. at 37·5° led to post-operative swelling of the perfused region from which recovery took place in 3–4 days. Higher concentrations led to immediate toxic changes during perfusions as previously described.

Perfusion with a total dose of 180 mg. of Chlorambucil in a 22 kg. dog at a concentration of 540 μg./ml. (Fig. 2) gave immediate destruction of the limb while a 23 kg. dog perfused with the same dose of Chlorambucil but with an increased circulatory volume giving a concentration of 275 μg./ml. had only mild post-operative oedema. In a 25 kg. dog use of 86 mg. of Chlorambucil at a concentration of 250 μg./ml. again led to only mild post-operative oedema of the perfused limb.

(2) *Temperature.*—The reaction velocity of Chlorambucil is doubled on raising the temperature 10°. That the reaction *in vivo* varies in a similar way is shown by the following experiments.

(*a*) A 22 kg. greyhound perfused with 180 mg. of Chlorambucil (concentration 540 μg./ml.) 37·5° C. suffered severe destruction of the limb in 10 minutes.

(*b*) A 25 kg. greyhound perfused with 130 mg. of Chlorambucil (concentration 400 μg./ml.) at 42° C. suffered even more rapid and complete necrosis.

On reducing the temperature of the arterial blood to 24° C. there was marked reduction in the drug uptake. At this temperature there was vascular spasm leading to reduction in capillary circulation.

(3) *Flow rate.*—Flow rates between 10–70 ml./minute were used and no gross difference in the rate of drug uptake occurred over this range.

(4) *Steroids.*—On two occasions during perfusion when signs of tissue damage were present 100 mg. of hydrocortisone was added to the perfusion fluid but nevertheless the limb progressed to necrosis.

(5) *Papaverine.*—This drug has a marked effect on arterial musculature, causing vascular dilatation and the opening up of arterio-venous shunts in the perfused region, thus reducing the circulation time. This effect might cause a partial by-pass of a tumour-bearing area and for this reason it seems inadvisable to use papaverine. The increase in arterial pressure which occurs on perfusing Chlorambucil occurs at a level of the drug which is toxic to the normal tissues and therefore should be avoided. Administration of papaverine when arterial pressure was high reduced this pressure as shown in Fig. 2, but did not prevent destruction of the tissues.

*Regional infusions*

All infusions were carried out at an arterial blood temperature of 37·5° C. In two experiments the technique of perfusion was carried out on the hind limb and the entire venous return was collected and not recirculated. Fresh blood containing Chlorambucil was twice added to the reservoir when the latter was nearly empty (Table I).

In all other infusions there was no occlusion of the venous return from the region. Chlorambucil and tagged erythrocytes were well mixed in the blood

TABLE I.—*Results of Attempts to Wash Out Chlorambucil that had been Absorbed by the Limb from the Extra-corporeal Circulation with Fresh Blood*

| Number of dog | 1 | 2 |
|---|---|---|
| Dog's weight | 23 kg. | 23 kg. |
| Chlorambucil contained by blood entering the limb (200 ml. over 10 min.) | 52 mg. | 54 mg. |
| Immediate return contained | 16 mg. | 7·5 mg. |
| 1st wash out contained 200 ml. | 3 mg. | 8·2 mg. |
| 2nd wash out contained 200 ml. | 1 mg. | — |
| Total Chlorambucil leaving limb | 20 mg. | 15·7 mg. |

used for infusion. If the assumption was made that the vascular distribution of the Chlorambucil and the tagged cells remained identical the percentage of Chlorambucil leaving the region may be calculated from :

$$\frac{\dfrac{\text{Drug conc. in vein draining region} - \text{conc. in systemic circulation}}{\text{Drug conc. in the infused blood}}}{\dfrac{\text{Counts/ml./sec. in vein draining region} - \text{counts/ml./sec. in systemic circulation}}{\text{Counts/ml./sec. in the infused blood}}} \times 100$$

= per cent of total dose leaving the region.

Two right femoral infusions were carried out on dogs weighing 22 kg. and 31 kg. respectively. Samples of venous blood were taken from the right femoral vein at regular intervals throughout the infusion. 60 mg. of Chlorambucil was given in each infusion and no toxic changes occurred in either case. The results were similar and are presented in Fig. 4. These results show that the average Chlorambucil return in the venous blood from the infused hind limb is approximately 50 per cent.

*Infusions of special regions*

(1) *Pelvic infusion.*—The right external iliac artery was cannulated while the aorta below the region of the inferior mesenteric was clamped as was also the left external iliac in a dog weighing 11·5 kg. Venous samples were obtained from the inferior vena cava and 45 mg. of Chlorambucil was administered. The results (Fig. 5) show that approximately 50 per cent of the amount of drug infused returned. A similar result was obtained on repeating the experiment.

(2) *Infusion of the right side of brain, head and neck.*—The right carotid artery of a 29 kg. Labrador was cannulated and infused with 60 mg. of Chlorambucil in 250 ml. of blood. During the infusion there was a profuse secretion of mucus from the right nostril and the character of the respiration changed, but no vasomotor changes occurred. The dog made a complete recovery. The results (Fig. 6) show that 60 per cent of the Chlorambucil given returned from the region into the systemic circulation.

In experiments in which dogs were given intravenous whole body doses of 4 mg./kg. body weight, severe leucopaenia with weakness, vomiting and diarrhoea always resulted by the seventh day. Doses of 2 mg./kg. caused no changes in general health or blood picture in dogs. It may be deduced that in cases of infusion where there is no sign of toxicity and no marrow depression, the amount of venous leak is less than 2 mg./kg. ; in cases of severe reaction then at least 4 mg./kg. has leaked into the general circulation.

The hind limbs of a 31 kg. Labrador were infused with a total dose of 120 mg. of Chlorambucil and resulted in no systemic ill effects, therefore the venous return would have been 60 mg. or less, i.e. 2 mg./kg., in agreement with the results shown in Fig. 4.

FIG. 4.—Hind limb infusion at 37·5° C. Broken lines indicate $^{51}Cr$ levels, continuous lines Chlorambucil concentrations ; ○—○, □—□ blood entering femoral artery, ●—●, △—△ in samples taken from the femoral vein ■—■ see text. Systemic samples gave 0·9, 1·35, 2·0, 2·2 counts/ml./sec. and had drug concentrations of 2, 2·5, 1·5, 2 μg./ml. at 10, 20, 30 and 40 min. respectively. Total dose 60 mg. Chlorambucil.

In another experiment a 21 kg. greyhound was given 120 mg. of Chlorambucil in 400 ml. by infusion of the femoral vein ; this led to evidence of mild systemic toxic signs—vomiting, loss of weight, depression in leucocytes and increased blood urea. From our findings of a 50 per cent venous return of Chlorambucil it may be estimated that this dog received into his systemic system 60 mg. (i.e. 50 per cent infused dose), which is 3 mg./kg., a dose which would be expected to give the above clinical picture.

### DISCUSSION

In a perfusion using a therapeutic dose of Chlorambucil (Fig. 1) there was rapid initial drop in concentration after which the levels in the arterial and venous blood were identical but slowly declined.

FIG. 5.—Pelvic infusion at 37·5° C. Brolen lines indicate $^{51}Cr$ levels, continous lines Chlorambucil concentrations; ○—○, ■—■ blood entering right external iliac artery, ●—●, △—△ blood from inferior vena cava, □—□, ×—× systemic levels. The percentage of drug leaving the region was determined as described in the text. Total dose 60 mg. Chlorambucil.

Part of the initial drop is due to dilution of the drug by the blood in the limb, as shown by a similar smaller drop in the concentration of the $^{51}Cr$-labelled erythrocytes. Complete mixing of the isolated circuit occurs in 5–10 minutes and allowing for the dilution 45–55 per cent of the Chlorambucil added to the reservoir has left or reacted in the vascular system during this time. *In vitro* experiments determining the rate at which Chlorambucil reacts in blood at 37·5° indicate that most of this is the result of absorption by the limb (Fig. 1).

When therapeutic doses are administered approximately 50 per cent of the

Chlorambucil leaves the blood in the first 5–10 minutes and since subsequently the arterial and venous lines are at the same concentration no more drug is absorbed by the limb. When destructive doses of Chlorambucil are used (Fig. 2) the drug level rapidly falls to undetectable levels. This may be due to breakdown of the capillary bed and subsequent uptake by the tissues.

Fig. 6.—Infusion of right side of head and neck at 37·5° C. Broken lines indicate $^{51}Cr$ level, continuous lines Chlorambucil concentration ; ○—○, ■—■ blood entering right carotid artery, ●—●, △—△ blood from right jugular veing, □—□ systemic $^{51}Cr$. The drug entering the systemic circulation was rapidly absorbed from the vascular system since the Chlorambucil levels were too low to be detected. The percentage of drug leaving the region was estimated as described in the text. Total dose 60 mg. Chlorambucil.

The results given by a normal perfusion (Fig. 1) seem to indicate that equilibrium is rapidly attained between the limb tissue and the blood. The data in Table I, however, suggest that there may be some mechanism, either in the cells or the vascular supply which prevents further absorption after the first 10 minutes of perfusion. There is, therefore, little advantage in prolonging the perfusion for more than 10–15 minutes and indeed it would seem likely that the slow infusion of Chlorambucil (up to twice the systemic dose at a concentration of approximately 200 $\mu$g./ml. at 37·5° ) into the main artery supplying the tumour-containing region would be the best method of administration. Direct arterial injection in the normal manner is not considered as satisfactory because of the destructive effects of a concentrated solution, the comparison between the limb damage caused by direct injection of one femoral artery and infusion of the other in one of the dogs being marked. Furthermore, occlusion of the artery will slow the regional blood flow and aid the local absorption of the drug.

The rapid initial absorption of Chlorambucil is probably connected with the nature of the group attached to the di-$\beta$-chloroethyl part of the molecule since Melphalan in which L-phenylalanine replaces phenylbutyric acid is absorbed slowly but throughout the entire perfusion (Fig. 3).

Several drugs are to be tested to determine the reason for the initial rapid absorption of Chlorambucil ; it may be due to the surface active properties of the drug. A knowledge of the chemical structures which will enable nitrogen mustards to penetrate tissues rapidly and to be held until reaction has occurred could be used to synthesise the alkylating agents most suitable for regional use.

### SUMMARY

The behaviour of Chlorambucil in experimental regional perfusions and infusions in the limbs of dogs has been studied in order to determine the probable optimum conditions for clinical use.

At 37·5° C. concentrations below 200 $\mu$g./ml. cause no damage. Levels between 250–300 $\mu$g./ml. cause mild reversible oedema, but with higher levels than this necrosis occurs. Increasing the temperature increases the activity of the drug.

The initial rapid tissue uptake of the drug in comparison with Melphalan is shown. Evidence is presented in support of use of Chlorambucil by intra-arterial infusion.

We wish to express our gratitude to the Council of the Royal College of Surgeons of England for permission to work at the Buckston-Browne Research Farm, and Mr. F. Watson for skilled technical help. Dr. E. Field kindly provided the labelled cells and Dr. W. Ross the sample of Chlorambucil.

One of us (M. D. S.) is in receipt of a Gordon Jacobs Fellowship.

This investigation has been supported by grants to the Chester Beatty Research Institute (Institute of Cancer Research : Royal Cancer Hospital) from the Medical Research Council, the British Empire Cancer Campaign, the Jane Coffin Childs Memorial Fund for Medical Research, the Anna Fuller Fund, and the National Cancer Institute of the National Institutes of Health, U.S. Public Health Service.

### REFERENCES

BARBERIO, J. R., KLOPP, C. T., AYRES, W. W. AND GROSS, H. A.—(1951) *Cancer*, **4**, 1341.

BRYANS, F. AND PYMAN, F. L.—(1929) *J. chem. Soc.*, p. 549.

CREECH, O., KREMENTZ, E. T., RYAN, R. F. AND WINBLAD, J. N.—(1958) *Ann. Surg.*, **148**, 616.

KLATT, O., GRIFFIN, A. C. AND STEHLIN, J. S.—(1960) *Proc. Soc. exp. Biol. N.Y.*, **104**, 629.

MOLLISON, P. L. AND VEALL, N.—(1955) *Brit. J. Haemat.*, **1**, 62.